# Augmented risk of ischemic stroke in hypertrophic cardiomyopathy patients without documented atrial fibrillation

**DOI:** 10.1038/s41598-022-19895-x

**Published:** 2022-09-22

**Authors:** You-Jung Choi, Bongseong Kim, Tae-Min Rhee, Hyun-Jung Lee, Heesun Lee, Jun-Bean Park, Seung-Pyo Lee, Kyung-Do Han, Yong-Jin Kim, Hyung-Kwan Kim

**Affiliations:** 1 Division of Cardiology, Department of Internal Medicine, Korea University Guro Hospital & Korea University College of Medicine, Seoul, Korea; 2 Department of Internal Medicine, Seoul National University College of Medicine, Seoul, Korea; 3 Department of Biostatistics, The Catholic University of Korea, Seoul, Korea; 4 Division of Cardiology, Department of Internal Medicine, Seoul National University Hospital, Seoul, Korea; 5 Healthcare System Gangnam Center, Seoul National University Hospital, Seoul, Korea; 6 Department of Statistics and Actuarial Science, Soongsil University, Seoul, Korea; 7 Section of Cardiovascular Imaging, Division of Cardiology, Department of Internal Medicine, Cardiovascular Center, Seoul National University Hospital & Seoul National University College of Medicine, 101 Daehak-ro, Jongno-gu, Seoul, 03080 Korea

**Keywords:** Cardiology, Risk factors

## Abstract

Although atrial fibrillation (AF) is a well-established risk factor for ischemic stroke (IS) in hypertrophic cardiomyopathy (HCM), the risk of IS in HCM patients without documented AF is less recognized. This nationwide population-based cohort study using Korean National Health Insurance database included 8,328 HCM patients without documented AF and 1:2 propensity score-matched 16,656 non-HCM controls between 2010 and 2016. The primary outcome was an incident IS. During a mean follow-up of 6.1 years, IS occurred in 328/8,328 (3.9%) patients with HCM and 443/16,656 (2.7%) controls. The overall incidence of IS was 0.72/100 person-years in the HCM group, which was significantly higher than that in the control group (0.44/100 person-years) (HR 1.64; 95% CI 1.424–1.895; *P* < 0.001). The overall incidence of IS was 1.36/100 person-years in HCM patients aged ≥ 65 and 2.32/100 person-years years in those with heart failure, respectively. In the HCM group, age ≥ 65 years (adjusted HR 2.74; 95% CI 2.156–3.486; *P* < 0.001) and chronic heart failure (adjusted HR 1.75; 95% CI 1.101–2.745; *P* = 0.018) were independent risk factors for IS. HCM patients without documented AF are at a greater risk of IS, especially in those 65 years of age or older or those with chronic heart failure.

## Introduction

Hypertrophic cardiomyopathy (HCM) is the most common genetic cardiomyopathy, characterized by left ventricular (LV) hypertrophy in the absence of abnormally increased loading conditions^1^. The clinical manifestations of HCM are variable, including angina, arrhythmia, thromboembolic events, heart failure, and sudden cardiac death^2^. Ischemic stroke is one of the most disastrous complications with high morbidity and mortality^3^, and its prevalence is approximately 10% in the general HCM population^4^.

A major risk factor for ischemic stroke is atrial fibrillation (AF), and the overall incidence rate of AF-associated ischemic stroke is 2.9% per year in patients with HCM^4^. Earlier studies have provided indisputable evidence of the benefits of anticoagulation for stroke prevention in HCM patients with AF^3,5^. Recent practice guidelines also strongly recommend oral anticoagulation therapy as a default treatment option regardless of the CHA_2_DS_2_-VASc score in HCM patients with persistent or paroxysmal AF^6^.

However, the overall burden of ischemic stroke in real-world clinical practice does not always result in the documentation of AF burden in patients with HCM^7–9^. This means that the current guideline is not sufficient for optimal risk stratification and management of HCM patients without documented AF. Moreover, the management guidelines for primary prevention of ischemic stroke in HCM patients without documented AF are still lacking. Therefore, this study aimed to determine the risk of ischemic stroke and identify its risk factors in HCM patients without documented AF.

## Results

### Study population

After propensity score (PS) matching with a 1:2 ratio, 8,328 patients with HCM and 16,656 controls were selected for the final analysis. All baseline characteristics were well-balanced regarding the demographic characteristics and comorbidities between the two groups (Table 1). The mean age was 57.4 ± 13.3 years, and 69.1% were males in the HCM group, whereas the mean age was 57.9 ± 12.7 years, and 68.8% were males in the control group.

**Table 1:** Baseline characteristics of the study population.

Variables	Before PS matching		ASD*	After PS matching		ASD*
	HCM group N = 10,572	Control group N = 86,700		HCM group N = 8,328	Control group N = 16,656	
**Age, years**	57.8 ± 13.5	55.7 ± 13.2	0.157	57.4 ± 13.3	57.9 ± 12.7	0.036
< 65 years	7191 (68.0)	64,678 (74.6)	0.146	5755 (69.1)	11,456 (68.8)	0.007
65–75 years	2173 (20.6)	15,117 (17.4)	0.080	1714 (20.6)	3668 (22.0)	0.035
≥ 75 years	1208 (11.4)	6905 (8.0)	0.117	859 (10.3)	1532 (9.2)	0.038
Male, n (%)	7149 (67.6)	60,978 (70.3)	0.059	5799 (69.6)	11,428 (68.6)	0.022
Low-income, n (%)	1843 (17.4)	17,026 (19.6)	0.568	1408 (16.9)	2750 (16.1)	0.011
**Comorbidities, n (%)**						
Chronic heart failure	2098 (19.8)	1179 (1.7)	0.630	197 (2.4)	239 (1.4)	0.068
Hypertension	5939 (56.2)	17,862 (20.6)	0.786	4326 (52.0)	8929 (53.6)	0.033
Diabetes mellitus	1417 (13.4)	6662 (7.7)	0.187	1080 (13.0)	2207 (13.3)	0.008
Dyslipidemia	4392 (41.5)	10,083 (11.6)	0.720	3123 (37.5)	6231 (37.4)	0.002
Vascular disease ^†^	761 (7.2)	3954 (4.6)	0.112	554 (6.7)	1089 (6.5)	0.005
ESRD	66 (0.6)	98 (0.1)	0.084	21 (0.3)	20 (0.1)	0.031
PM implanted, n (%)	13 (0.1)	8 (0.01)	0.044	2 (0.02)	3 (0.02)	0.004

*ASD* absolute standardized difference, *ESRD* end-stage renal disease, *HCM* hypertrophic cardiomyopathy, *PM* pacemaker, *PS* propensity score.

*ASD of < 0.1 indicated a negligible difference between the two groups.

^†^ Vascular disease included myocardial infarction and peripheral artery disease.

### Primary outcome

**Figure 1:**
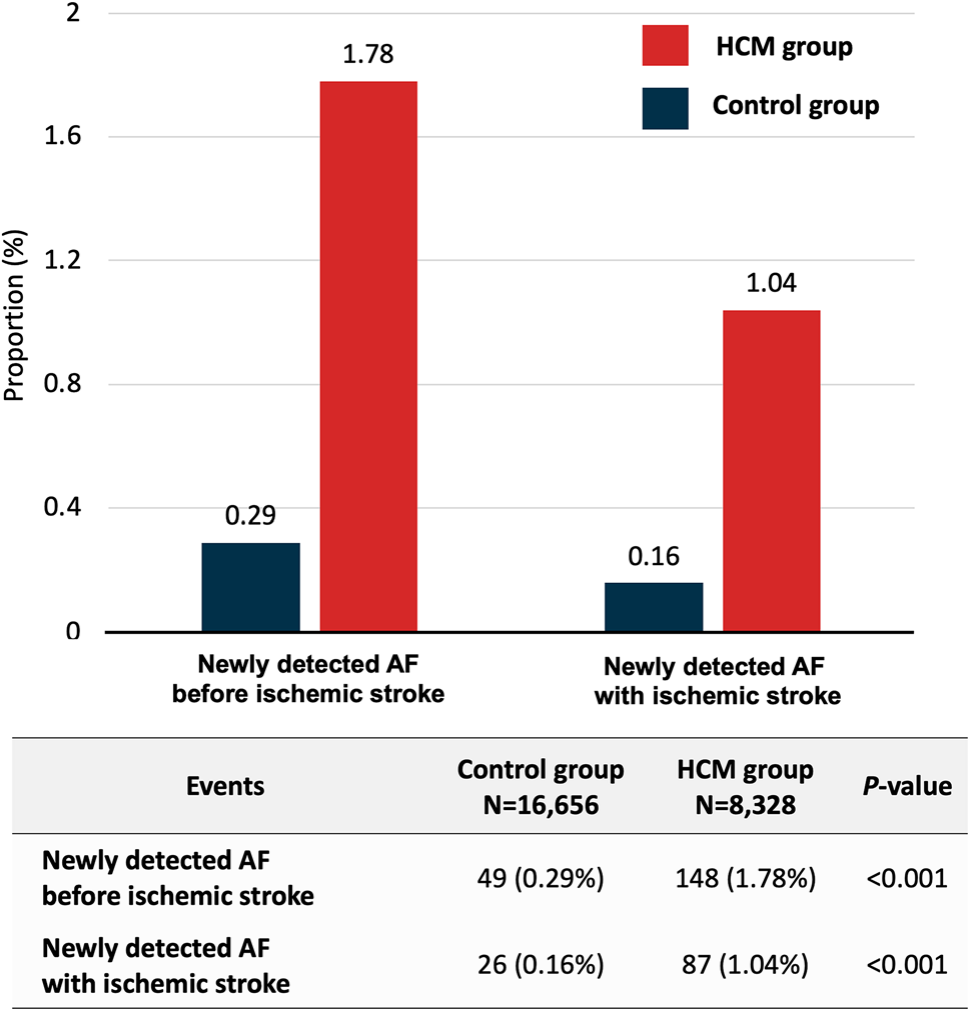
Newly detected atrial fibillation (AF) before or concomitantly with ischemic stroke. Newly detected AF before ischemic stroke was more frequent in the hypertrophic cardiomyopathy (HCM) group than in the non-HCM control group. Newly detected AF concomitantly with ischemic stroke was also more frequently observed in the HCM group than in the control group.

The overall mean follow-up was 6.1 ± 2.4 years and 5.5 ± 2.6 years in the HCM and control groups, respectively. During the follow-up, censored cases due to newly detected AF before the onset of the ischemic stroke (148/8,328 [1.78%] vs. 49/16,656 [0.29%], *P* < 0.001) and AF detected concomitantly with the incident ischemic stroke (87/8,328 [1.04%] vs. 26/16,656 [0.16%], *P* < 0.001) were also more common in the HCM group (Fig. 1).

**Figure 2:**
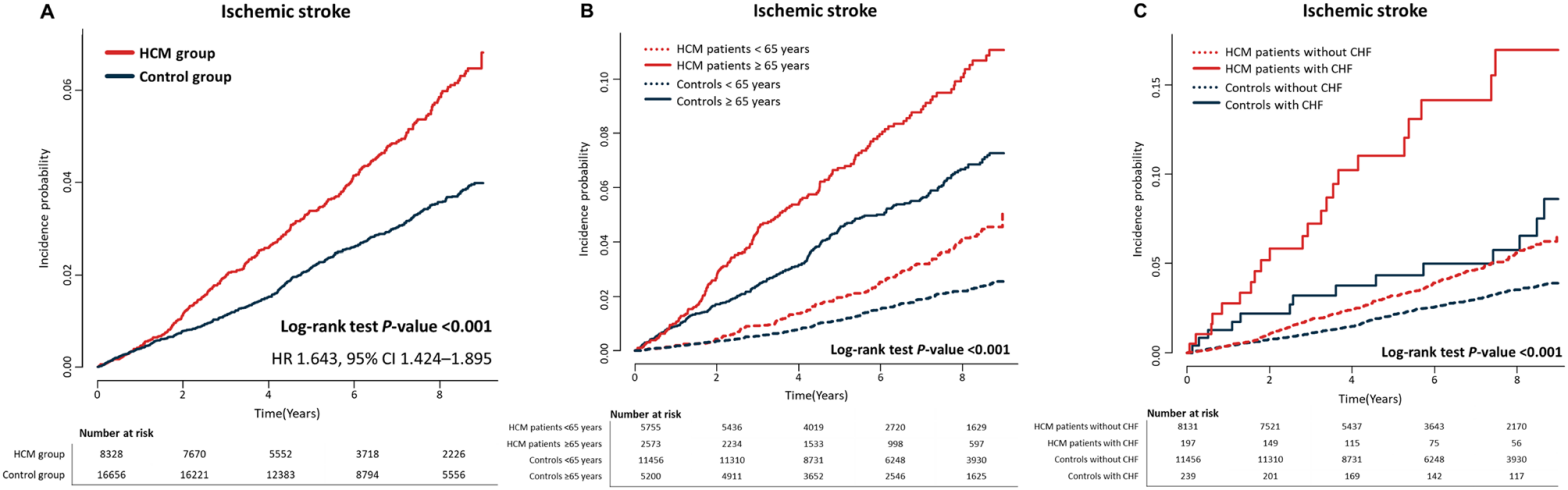
Kaplan–Meier curve showing incident ischemic stroke. (**A**) Kaplan–Meier curve for incident ischemic stroke in the hypertrophic cardiomyopathy (HCM) and control groups. Kaplan–Meier curves for incident ischemic stroke stratified by (**B**) age (< 65 and ≥ 65 years) and (**C**) the presence or absence of chronic heart failure (CHF). Neither group had documented atrial fibrillation nor a previous history of thromboembolic events, anticoagulation use, or cancer.

Incident ischemic stroke was observed in 328 of 8,328 (3.9%) and 443 of 16,656 (2.7%) individuals in the HCM and control groups, respectively. Among individuals who developed ischemic stroke, the proportion of concomitantly detected AF accounted for 26.5% (87/328) and 5.8% (26/443) in the HCM and control groups, respectively. The overall incidence of ischemic stroke was 0.72/100 person-years in the HCM group, which was significantly higher than that in the control group (0.44/100 person-years; hazard ratio [HR] 1.64; 95% confidence interval [CI], 1.424–1.895; *P* < 0.001). The Kaplan–Meier curve demonstrated that the HCM group had a higher incidence probability of ischemic stroke than the control group (log-rank test, *P* < 0.001) (Fig. 2A).

### Subgroup analysis

In the subgroup analysis according to age (< 65 years vs. ≥ 65 years), sex, and the presence or absence of comorbidities (chronic heart failure, hypertension, dyslipidemia, and vascular disease), the risk of ischemic stroke was consistently higher in the HCM group than in the control group (Online Supplementary Table S1). Meanwhile, among patients without diabetes mellitus, the risk of ischemic stroke was significantly higher in the HCM group than in the control group (adjusted HR 1.80, 95% CI 1.532–2.109; *P* < 0.001), whereas no statistically significant difference was observed in the presence of diabetes mellitus between two groups (HR 1.17, 95% CI 0.845–1.621; *P* = 0.736) (*P* for interaction = 0.004).

### Risk factors for ischemic stroke

Compared to patients with HCM without incident ischemic stroke, those who developed ischemic stroke were older (mean age, 64.8 ± 11.6 vs. 57.2 ± 13.3; *P* < 0.001) and had more comorbidities, including chronic heart failure (6.7% vs. 2.2%, *P* < 0.001), hypertension (64.3% vs. 51.4%, *P* < 0.001), and diabetes mellitus (16.8% vs. 12.8%, *P* = 0.037) (Online Supplementary Table S2). After adjusting for sex, low income, chronic heart failure, hypertension, diabetes mellitus, dyslipidemia, vascular disease, and pacemaker implantation, patients with HCM aged ≥ 65 years had a 2.7-fold higher risk of ischemic stroke than those aged < 65 years (95% CI 2.156–3.486; *P* < 0.001). In addition, a history of chronic heart failure remained an independent risk factor for ischemic stroke, which was associated with a 1.7-fold higher hazard (95% CI 1.101–2.745; *P* = 0.018) (Table 2).

**Table 2:** Cox regression analysis for the risk of ischemic stroke in patients with hypertrophic cardiomyopathy without documented atrial fibrillation.

Variables	Univariate analysis	*P*-value	Multivariate analysis*	*P*-value
	HR (95% CI)		HR (95% CI)	
Age, ≥ 65 years	3.01 (2.420–3.737)	< 0.001	2.74 (2.156–3.486)	< 0.001
Sex, Female	1.29 (1.024–1.613)	0.031	0.80 (0.624–1.018)	0.070
Hypertension	1.69 (1.350–2.121)	< 0.001	1.18 (0.925–1.494)	0.185
Diabetes mellitus	1.44 (1.079–1.926)	0.013	1.02 (0.751–1.378)	0.913
Chronic heart failure	3.40 (2.206–5.241)	< 0.001	1.74 (1.101–2.745)	0.018
Vascular disease ^†^	1.49 (1.019–2.184)	0.040	0.97 (0.652–1.436)	0.871

*CI* confidence interval, *HR* hazard ratio.

*Adjusted for age, sex, low income, chronic heart failure, hypertension, diabetes mellitus, dyslipidemia, vascular disease (including myocardial infarction and peripheral artery disease), and pacemaker implantation.

^†^Vascular disease included myocardial infarction and peripheral artery disease.

**Figure 3:**
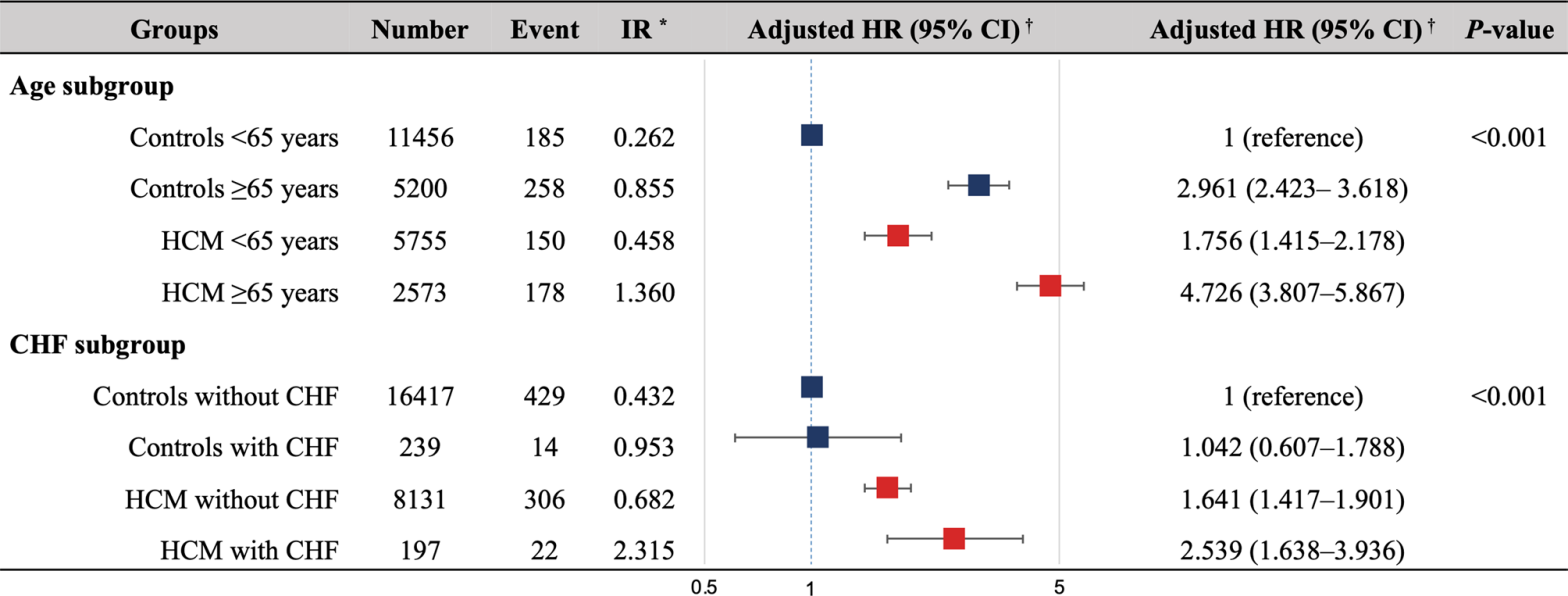
Incidence rate and adjusted hazard ratio of ischemic stroke. Incidence rate and adjusted hazard ratio of ischemic stroke in the hypertrophic cardiomyopathy (HCM) and control groups according to age and chronic heart failure (CHF). The overall incidence rate of ischemic stroke was 1.36 per 100 person-years and 2.32 per100 person-years in patients with HCM aged ≥ 65 years and those with CHF, respectively. Besides, the adjusted hazard ratio (HR) for ischemic stroke was 4.7 and 2.5 in patients with HCM aged ≥ 65 years and those with CHF, respectively. *Incidence rate (IR) was presented as 100 person-years. ^†^Adjustment for age, sex, low income, CHF, hypertension, diabetes mellitus, dyslipidemia, vascular disease (including myocardial infarction and peripheral artery disease), and pacemaker implantation. CI, confidence interval.

To further investigate the impact of age and chronic heart failure on the incident ischemic stroke, the HCM and control groups were stratified according to age (< 65 years vs. ≥ 65 years) and the presence or absence of chronic heart failure. The overall incidence of ischemic stroke was 1.36/100 person-years and 2.32/100 person-years in HCM patients aged ≥ 65 years and those with chronic heart failure, respectively. Also, adjusted HR for ischemic stroke was 4.73 (95% CI 3.807–5.867) and 2.54 (95% CI 1.638–3.936) in HCM patients aged ≥ 65 years and those with chronic heart failure, respectively (Fig. 3). The Kaplan–Meier curves demonstrated that HCM patients aged ≥ 65 years had the highest risk of ischemic stroke compared to those aged < 65 years and the control group (log-rank test, *P* < 0.001) (Fig. 2B). Similarly, HCM patients with chronic heart failure had the highest risk of ischemic stroke compared to those without chronic heart failure and the control group (log-rank test, *P* < 0.001) (Fig. 2C).

## Discussion

In this large-scale nationwide population-based cohort study, we observed a 1.6-fold higher hazard of ischemic stroke in HCM patients without documented AF compared to the control group. A higher risk of ischemic stroke was consistently observed in almost all subgroups. The only exception was regarding diabetes mellitus, where the risk of ischemic stroke was significantly increased only in HCM patients without diabetes mellitus, compared to the non-HCM control group. Notably, the two most significant predictors for ischemic stroke were older age, especially ≥ 65 years, and the presence of chronic heart failure in HCM patients without documented AF.

AF is the most frequently encountered arrhythmia in patients with HCM, with an overall prevalence of 27% and an annual incidence of 3.8%^10^. The risk of thromboembolic events in HCM has been recognized as clinically important, especially in documented AF; thus, the effectiveness and efficiency of anticoagulation strategies are well established in HCM patients with documented AF^7,11,12^. Given the clinical implication of documented AF in patients with HCM, the current guidelines for preventing and managing thromboembolic risk predominantly focus on HCM patients with documented AF^6^. However, as they constitute only 20% of the HCM population, 80% of the remaining patients with HCM remain neglected because AF is yet to be documented^4^. In fact, as demonstrated in our study, HCM patients without documented AF are not entirely free from the risk of ischemic stroke, and some of them are clearly at higher risk than the PS-matched control population.

In this study, patients with HCM had an increased risk of ischemic stroke, even in the absence of documented AF. We observed that approximately 3.9% of the HCM patients without documented AF experienced an ischemic stroke during 6.1 years of follow-up, which was a 1.6-fold higher risk compared to the control group. Similarly, in a previous nationwide cohort study in Taiwan, the overall incidence of ischemic stroke was approximately 4.9% in HCM patients without AF during a median follow-up period of 7.3 years^8^. Thus, the number of patients with HCM initially deemed to be at low risk of ischemic stroke cannot be neglected. In addition, our findings demonstrated that a large proportion (26.5%) of HCM patients with ischemic stroke turned out to have AF at the time of ischemic stroke. This leads us to speculate that many patients in the HCM cohort had atrial dysfunction including undetected AF before the ischemic stroke and that early detection of AF has an advantage in preventing ischemic stroke.

Since the contemporary prevention strategies targeting ischemic stroke have significantly improved^5,6^, it is time to shift our interest and focus on the majority of HCM patients without documented AF who are at high risk for ischemic stroke. Therefore, identifying significant predictors is a cornerstone of managing the risk of ischemic stroke in this unattended HCM population.

In real-world clinical practice, it is not practical to monitor every HCM patient for potential risk of ischemic stroke or prescribe prophylactic anticoagulants in HCM patients without documented AF. Although patients with HCM have an increased risk of ischemic stroke compared to the general population, the annual incidence of ischemic stroke in patients with HCM is reported to be less than 1%^11^, and the incidence of ischemic stroke in HCM patients without documented AF is not well-known. We also observed that the overall incidence of ischemic stroke was 0.7% per person-year in HCM patients without documented AF, which was not high enough to justify anticoagulation therapy in every HCM patient^13^. On the other hand, our findings demonstrated that even without a documented history of AF, patients with HCM had a significantly higher risk for ischemic stroke if they were aged ≥ 65 years or had a diagnosis of chronic heart failure. The estimated incidence rate of ischemic stroke was 1.36% per person-year and 2.32% per person-year for patients with HCM aged ≥ 65 years and for those with chronic heart failure, respectively, exceeding 1% per person-year; therefore, active preventive strategies are required^13^.

“Old age” is the most potent non-modifiable risk factor for ischemic stroke in the general population, with approximately three-quarters of all strokes occurring in people aged ≥ 65 years^14^. Given that older patients with HCM are more likely to have subclinical AF, it is recommended that they undergo 48-h ambulatory electrocardiogram monitoring every 6–12 months to detect AF^15^. In this regard, similar to our observations, a previous study reported that HCM patients aged ≥ 65 years had a 1.3-fold higher risk of ischemic stroke than those aged < 65 years, even when AF was not documented^8^. Hence, considering that older patients with HCM are prone to experience cardiovascular complications other than sudden cardiac death^16^, effective prevention and early interventions are expected to have greater benefits to improve their prognosis by preventing ischemic stroke in older HCM patients without documented AF^13^.

Chronic heart failure is a definitive indication for anticoagulation therapy to reduce thromboembolic risk in patients with AF at any age^17^. In patients with HCM, heart failure-related dyspnea is reported to be associated with an increased risk of thromboembolic events^11,18^. In the current study, we demonstrated here that chronic heart failure in patients with HCM was closely related to an increased risk of ischemic stroke, even without documented AF. Although the underlying mechanisms of ischemic stroke development in patients with HCM are fully understood, the end-stage HCM, defined as HCM with an LV ejection fraction of < 50%, has been reported to be linked to the increased risk of ischemic stroke^19^. While LV hypertrophy exacerbates left atrial thrombus formation in relation to atrial arrhythmia, frequent mural thrombus formation in the end-stage HCM is reported to cause thromboembolic events, even in sinus rhythm^19^. In addition, LV diastolic impairment could lead to left atrial dilation, which could responsible for the occurrence of AF^20^. Although a direct demonstration of this possibility is still lacking, there is also speculation that thrombus formation of the endothelial cell is induced by dynamic LV outflow tract obstruction in HCM^21^. Therefore, although previous randomized studies on oral anticoagulation for heart failure in sinus rhythm reported disappointing results in a prevention context^22,23^, the current study suggests a need for further randomized trials in high-risk populations such as HCM patients without documented AF.

Of note, the association between patients with HCM and the increased risk of ischemic stroke differed depending on the presence or absence of diabetes mellitus. Diabetes mellitus is one of the most important risk factors for thromboembolic events, and unsurprisingly, HCM patients with diabetes mellitus are older and have stroke-prone cardiovascular risk profiles^24^. Therefore, it is reasonable to interpret that the impact of HCM on incident ischemic stroke might be attenuated because the clinical characteristics of the two groups became similar in favor of the occurrence of an ischemic stroke. Further studies on the pathophysiologic mechanisms in HCM patients with diabetes mellitus for the development of ischemic stroke are needed.

Despite recent advances in therapeutic strategies for HCM-related complications, the incidence of ischemic stroke is still high; thus, it is a significant threat to contemporary patients with HCM^25^. Given the safety and effectiveness of oral anticoagulants in preventing ischemic stroke in patients with HCM and documented AF in a primary prevention setting^5,26^, its role in preventing ischemic stroke in HCM patients without documented AF remains to be evaluated.

### Limitation

First, this was a retrospective observational cohort study and was inevitably subjected to residual confounding. Second, this study utilized the NHIS claims database, which lacks the results of echocardiography imaging or laboratory tests. Thus, information on the morphological type of HCM or genetic mutations was unavailable. In addition, some brief episodes of AF could remain undetected as a clinical outcome because ambulatory electrocardiogram monitoring might have not been performed in all patients. Finally, although we suggested that chronic heart failure is a risk factor for ischemic stroke, we could not provide additional information regarding the severity of LV systolic function or the type and severity of heart failure-related symptoms, which were not available in the National Health Insurance Service (NHIS) database.

## Conclusion

HCM patients without documented AF are at a greater risk of developing ischemic stroke than the PS-matched control population; specifically, HCM patients aged ≥ 65 years and those with chronic heart failure are at an increased risk of ischemic stroke. Additional studies are required to confirm our findings and establish effective management strategies to prevent ischemic stroke in the majority of HCM patients without documented AF.

## Methods

### Study design and database

This nationwide population-based cohort study was conducted using the NHIS database. The NHIS is a mandatory social health insurance service that provides comprehensive medical care and covers almost the entire Korean population^27^. The NHIS database contains the longitudinal data of individuals, comprising sociodemographic information, medical billing, and claims submitted by medical institutions, including medical history^27^. Each individual's information was classified according to the *International Classification of Disease, Tenth Revision* (ICD-10) codes.

As part of the NHIS, the Korean government launched the *Rare Intractable Disease* program that offers financial support covering 90% of all medical expenses. Comprehensive reviews are undertaken for approval of the final registration for the *Rare Intractable Disease* program by qualified medical experts, and health insurance claims professionals, based on the imaging and laboratory test results and clinical information. This study was conducted following the ethical guidelines of the Declaration of Helsinki and was approved by the institutional review board of Seoul National University Hospital. The NHIS database contains fully de-identified personal information; thus, the need for informed consent was waived.

### Study population

HCM was defined by (i) at least one admission/outpatient clinic visit with the ICD-10 codes of I42.1/I42.2 and (ii) registration in the Korean *Rare Intractable Disease* program^4,5,28–31^. Previously, we validated this HCM definition by reviewing the medical records of 1100 patients, which confirmed a sensitivity and specificity of 91.5% and 100% for this definition, respectively^4^. A total of 19,372 patients with HCM were identified from the NHIS database between January 2010 and December 2016. We excluded patients aged ≤ 18 years; those with a record of AF (ICD-10 codes I480-484, I489) 3 years before and 1 year after the first diagnosis of HCM; those with a previous history of thromboembolic events, including ischemic stroke, transient ischemic attack, and arterial thromboembolism; those with a previously confirmed cancer, undergoing anticoagulation therapy, and those with missing variables (Online Supplementary Fig. S1).

The comparison group was selected from the same database using the same criteria. It comprised individuals from the general population without a previous history of documented AF, thromboembolic events, cancer, and anticoagulation therapy. Patients with HCM had a 1:2 PS matched by covariates (Table 1; please see the statistical method section for more details) to the non-HCM control participants (comparison group) for final analysis (Online Supplementary Fig. S1).

### Primary outcome

The primary outcome was an incident ischemic stroke defined using ICD-10 codes of I63 and I64 during hospitalization when the diagnostic code was registered with concomitant brain imaging studies^4,5^. The study cohort was followed up from the initial date of HCM diagnosis until the occurrence of thromboembolic events, AF before thromboembolic events, death, or the end of the study period (December 31, 2018), whichever came first. Death, AF development without the incident ischemic stroke, or emigration (withdrawal from the insurance program) before the primary outcome was considered a censored observation.

### Covariates

We collected information regarding the covariates relevant to the present study, including demographics (age, sex, and income level), traditional cardiovascular risk factors (i.e., chronic heart failure, hypertension, diabetes mellitus, dyslipidemia, myocardial infarction/peripheral artery disease, and end-stage renal disease), and history of pacemaker implantation. AF detected within one week from the diagnosis of ischemic stroke was regarded as AF diagnosed concurrently with ischemic stroke. Chronic heart failure was defined as a history of hospitalization for heart failure and/or at least two medical recodes of outpatient clinic visits with ICD-10 codes of I50, I42.0, I11.0, I13.0, and I13.2^29^. The specific definitions of the comorbidities used here are listed in Online Supplementary Table S3. These definitions have been validated previously^28–31^.

### Statistical analysis

Data were presented as mean ± standard deviation or median (interquartile range) for continuous variables and as numbers (percentages) for categorical variables. Categorical variables were compared using the χ^2^ test or Fisher's exact test, and continuous variables were compared using the Student's *t*-test or Wilcoxon's signed rank-sum test.

To address the potential differences in baseline characteristics, we used PS matching analysis, which has been widely applied to control all measurable confounders simultaneously. Specifically, a PS was calculated for all the participants using a logistic regression fit for HCM, adjusting for clinically relevant covariates, including demographics, comorbidities, and pacemaker implantation, as listed in Table 1. Each HCM patient was then matched to two non-HCM control participants with a caliper for the nearest-neighbor matching within the first four to eight digits. To examine the matching effectiveness, we computed absolute standardized differences, and a value less than 10% and closer to zero demonstrated that the variable was in a more balanced state between the two groups. Because two controls per HCM case were individually matched using PS, we used the date of diagnosis as the index date for both patients with HCM and matched controls.

The incidence rate was calculated as the sum of all the new episodes of the outcomes divided by the total follow-up duration in person-years. The incidence probability of the outcomes was plotted using the Kaplan–Meier curves with a statistical comparison using the log-rank test. Cox regression analysis was used to assess the effect of risk factors on event-free survival time by estimating the HRs with corresponding 95% CIs. Multivariate analysis was applied to estimate adjusted HRs and 95% CI for subgroup analysis after adjusting for all covariates used to balance the two groups during the PS matching, such as sex, low income, chronic heart failure, hypertension, diabetes mellitus, dyslipidemia, vascular disease, and pacemaker implantation.

SAS software (version 9.4; SAS Institute Inc., Cary, NC, USA) was used for all statistical analyses, and a *P*-value of < 0.05 was considered statistically significant.

## Supplementary Information

Supplementary Information.

## Data Availability

All data created and/or used during this study are not publicly available as the researchers who submit a separate application can only access the National Health Information Database of Korea NHIS only in designated places according to NHIS policy. Applications could be submitted through the NHIS website (https://nhiss.nhis.or.kr).
