# Supplementary material for: Augmented risk of ischemic stroke in hypertrophic cardiomyopathy patients without documented atrial fibrillation
Source: Sci Rep. 2022 Sep 22;12:15785. doi: 10.1038/s41598-022-19895-x (PMC9499955; doi:10.1038/s41598-022-19895-x)
Supplement: Supplementary file 1 — Supplementary Information. [file 41598_2022_19895_MOESM1_ESM.pdf]

Supplemental materials

Title: Augmented risk of ischemic stroke in hypertrophic cardiomyopathy patients without documented atrial fibrillation

You-Jung Choi, M.D., Ph.D.,[1,2] Bongseong Kim,[3] Tae-Min Rhee, M.D.,[2,4] Hyun-Jung Lee, M.D., [2,4] Heesun Lee, M.D.,[2,5] Jun-Bean Park, M.D., Ph.D., [2,4] Seung-Pyo Lee, M.D., Ph.D., [2,4] Kyung-Do Han, Ph.D.,[6] Yong-Jin Kim, M.D., Ph.D., [2,4] Hyung-Kwan Kim, M.D., Ph.D.[2,4]

[1] Division of Cardiology, Department of Internal Medicine, Korea University Guro Hospital, Seoul, Korea; Division of Cardiology; [2] Department of Internal Medicine, Seoul National University Hospital, Seoul, Korea; [3] Department of Biostatistics, The Catholic University of Korea, Seoul, Korea; [4] Department of Internal Medicine, Seoul National University College of Medicine, Seoul, Korea; [5] Healthcare System Gangnam Center, Seoul National University Hospital, Seoul, Korea; [6] Department of Statistics and Actuarial Science, Soongsil University, Seoul, Korea.

Address for correspondence:
Hyung-Kwan Kim, MD, Ph.D.
Professor and Director of Cardiac Diagnostic Test Unit
Section of Cardiovascular Imaging, Division of Cardiology, Cardiovascular Center, Seoul National University Hospital
Department of Internal Medicine, Seoul National University College of Medicine
101 Daehak-ro, Jongno-gu, Seoul 03080, Korea
Fax: +82-2-762-9622, Tel: +82-2-2072-0243
Email: cardiman73@gmail.com or hkkim73@snu.ac.kr

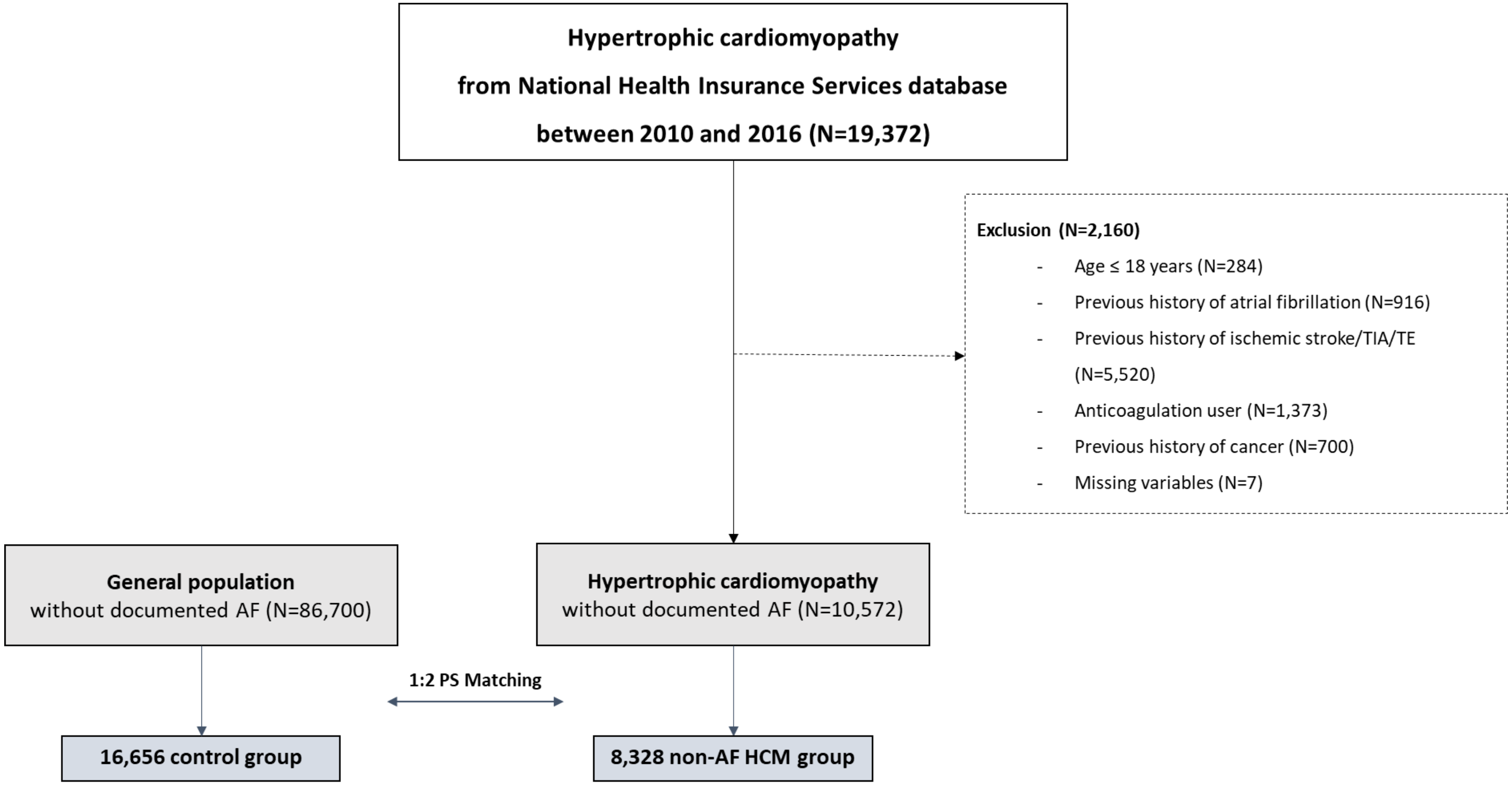

Figure S1. Flow chart of the study design

After exclusion of patients who had a previous history of atrial fibrillation, thromboembolic events, any diagnosis of cancer, or anticoagulation therapy, patients with hypertrophic cardiomyopathy (HCM) without documented atrial fibrillation (AF) were 1:2 propensity score (PS) matched to the non-HCM controls. TIA, transient ischemic attack; TE, arterial thromboembolism.

Table S1. Risk of incident ischemic stroke among subjects without documented atrial fibrillation in prespecified subgroups.

Subgroup	HCM group			Control group			Adjusted HR (95% CI) †	*P*-value	*P* for interaction
	Number	Event	IR *	Number	Event	IR *			
**Total**	8328	328	0.716	16656	443	0.439	1.643 (1.424–1.895)	<0.001	-
**Sex group**									
Male	5799	214	0.662	11428	270	0.390	1.742 (1.455–2.085)	<0.001	0.509
Female	2529	114	0.846	5228	173	0.549	1.553 (1.225–1.970)	<0.001	
**Age group**									
<65 years	5755	150	0.458	11456	185	0.262	1.876 (1.512–2.329)	<0.001	0.236
≥65 years	2573	178	1.360	5200	258	0.855	1.531 (1.263–1.855)	<0.001	
**Chronic heart failure**									
No	8131	306	0.682	16417	429	0.432	1.593 (1.376–1.845)	<0.001	0.236
Yes	197	22	2.315	239	14	0.953	2.401 (1.224–4.708)	<0.001	
**Hypertension**									
No	4002	117	0.527	7727	136	0.291	1.780 (1.388–2.282)	<0.001	0.484
Yes	4326	211	0.893	8929	307	0.569	1.609 (1.349–1.919)	<0.001	
**Diabetes mellitus**									
No	7248	273	0.680	14449	336	0.382	1.798 (1.532–2.109)	<0.001	0.004
Yes	1080	55	0.972	2207	107	0.837	1.170 (0.845–1.621)	0.736	
**Dyslipidemia**									

No	5205	192	0.663	10425	261	0.411	1.621 (1.346–1.954)	<0.001	0.952
Yes	3123	136	0.807	6231	182	0.488	1.678 (1.343–2.095)	<0.001	
**Vascular disease** ‡									
No	7774	299	0.695	15567	410	0.434	1.614 (1.391–1.874)	<0.001	0.557
Yes	554	29	1.026	1089	33	0.514	2.031 (1.232–3.347)	<0.001	

* IR was presented as 100 person-years.

† Adjusting for age, sex, low-income, chronic heart failure, hypertension, diabetes mellitus, dyslipidemia, vascular disease (including myocardial infarction and peripheral artery disease), and pacemaker implantation.

‡ Vascular disease included myocardial infarction and peripheral artery disease.

CI, confidence interval; HCM, hypertrophic cardiomyopathy; HR, hazard ratio; IR, incidence rate.

Table S2. Baseline characteristics of HCM patients with and without incident ischemic stroke

Variables	Incident ischemic stroke (-) N=8000	Incident ischemic stroke (+) N=328	*P*-value
Age, years	57.2±13.3	64.8±11.6	<0.001
<65	5605 (70.1)	150 (45.7)	<0.001
65–75	1609 (20.1)	105 (32.0)	
≥75	786 (9.8)	73 (22.3)	
Men, n (%)	5585 (69.8)	214 (65.2)	0.078
Low-income, n (%)	1340 (16.8)	68 (20.7)	0.059
Comorbidities, n (%)			
Chronic heart failure	175 (2.2)	22 (6.7)	<0.001
Hypertension	4115 (51.4)	211 (64.3)	<0.001
Diabetes mellitus	1025 (12.8)	55 (16.8)	0.037
Dyslipidemia	2987 (37.3)	136 (41.5)	0.130
Vascular disease *	525 (6.6)	29 (8.8)	0.105
ESRD	20 (0.3)	1 (0.3)	0.846
PM implanted, n (%)	2 (0.03)	0 (0.0)	0.775

* Vascular disease included myocardial infarction and peripheral arterial disease

AF, atrial fibrillation; ESRD, end-stage renal disease; HCM, hypertrophic cardiomyopathy; PM, pacemaker.

Table S3. Definitions of covariates

Covariates	ICD-10 codes or procedure/device code	Additional definitions
Chronic heart failure	I50, I42.0 (Dilated cardiomyopathy), I11.0, I13.0, I13.2	Admission ≥1 or outpatient clinic ≥2
Hypertension	I10-I13, I15	Admission ≥1 or outpatient clinic ≥2 and minimum 1 prescription of anti-hypertensive drug
Diabetes mellitus	E11-E14	Admission ≥1 or outpatient clinic ≥2 and minimum 1 prescription of anti-diabetic drugs
Dyslipidemia	E78	Admission or outpatient department≥1
Myocardial infarction	I21, I22	Admission ≥1 or outpatient clinic ≥2
Peripheral arterial disease	I70.2, I73	Admission ≥1 or outpatient clinic ≥2
End-stage renal disease	N18.5, Z49	Dialysis ≥ 2 (Procedure codes) O7011-7020 (HD), O7017, O7075 (PD)
Cancer	C00-97	Admission ≥1 or outpatient clinic ≥2
Pacemaker implantation	(Procedure code and Device code) O0203 + G8201/8202, O0204 + G8203/8204/8205,O0205 + G8201/8202, O0206/0207 + G8203/8204/8205	-

ICD-10, international Classification of Disease, Tenth Revision; HD, hemodialysis; PD, peritoneal dialysis.
